# Cross‐Sectional Survey of Critical Care Provision in Ministry of Health Hospitals in Zambia

**DOI:** 10.1111/nicc.70685

**Published:** 2026-09-24

**Authors:** Richard Kahalu, Joy Notter, Chris Carter

**Affiliations:** ^1^ Zambia Air Force Lusaka Zambia; ^2^ Birmingham City University Birmingham UK

## Abstract

**Background:**

Critical care is an essential component of universal health care; however, its provision in low‐ and middle‐income countries remains poorly understood. Although Zambia has expanded critical care services over the last 15 years, national evidence regarding workforce capacity, infrastructure and service provision remains limited.

**Aims:**

To evaluate critical care workforce, service provision and patient case mix across Ministry of Health hospitals in Zambia.

**Study Design:**

A cross‐sectional survey was administered across 14 public hospitals with critical care units across Zambia, representing all 10 provinces. Data were collected via an e‐survey questionnaire instrument. Descriptive statistical analysis was conducted using SPSS. Ethics approval was obtained prior to the study.

**Results:**

All hospitals responded (*n* = 14/14, 100% response rate), accounting for a capacity of 131 critical care beds. Only 28.6% of hospitals had 24‐h intensivist coverage, whereas all had registered critical care nurses. Equipment availability varied, with universal access to ventilators and monitors but limited access to syringe pumps and inconsistent availability of functional CT scanners. Over a 7‐day period, 121 admissions were recorded, predominantly medical (29%), surgical (24.8%) and trauma‐related (19.8%). Paediatric cases accounted for 40% of admissions. Formal admission and discharge protocols were present in fewer than half of the hospitals.

**Conclusion:**

The study provides the first national overview of public critical care provision in Zambia and provides a snapshot of current provision. The findings highlight urgent areas for capacity strengthening.

**Relevance to Clinical Practice:**

Findings are similar to other studies within the sub‐Saharan region and highlight the need to strengthen workforce training, particularly in critical and paediatric care, improve access to essential equipment and standardise care protocols to enhance patient safety and outcomes. The study demonstrates the importance of strengthening the multidisciplinary critical care workforce.

## Impact Statements

- What is known about the topic
  ○ Critical care is a key component of universal health coverage and is essential for improving patient outcomes and health system resilience.
  ○ Low‐ and middle‐income countries, particularly in sub‐Saharan Africa, face persistent challenges in critical care delivery, including limited infrastructure, workforce shortages and inadequate equipment.
  ○ Access to critical care services is often inequitable, with services concentrated in urban centres and limited availability of beds and essential resources.
  ○ Limited comprehensive national data in many low‐resource countries make it difficult to evaluate service provision, identify gaps and guide policy development.
- What this paper adds
  ○ Provides the first national cross‐sectional snapshot of critical care provision across Ministry of Health hospitals in Zambia.
  ○ Quantifies national capacity, identifying 131 critical care beds across 14 hospitals serving a large population, highlighting limited capacity relative to need.
  ○ Highlights significant workforce and service challenges, including limited 24‐h intensivist coverage; however, all units have the presence of registered critical care nurses.
  ○ Highlights variability and limitations in infrastructure and equipment, including inconsistent access to functioning CT scanners, syringe pumps and other essential resources.
  ○ Establishes a baseline dataset to inform future research, workforce planning and policy development aimed at strengthening critical care services in Zambia.

## 1 Introduction

Critical care services have been identified as an essential component of universal healthcare coverage, contributing to improved patient outcomes and healthcare resilience [1]. Zambia is a landlocked low‐ and middle‐income country (LMIC) in sub‐Saharan Africa with an estimated population of over 22 million, the majority of whom reside in rural areas [2]. For administration purposes, the country is divided into 10 provinces. Over the past 15 years, the Ministry of Health (MOH) has been gradually expanding critical care services, in terms of workforce, equipment and capacity [3, 4]. However, as with many low‐resource countries, the COVID‐19 pandemic also exposed persistent gaps in infrastructure, workforce availability and service delivery [5].

In the light of these changes, it is a cause for concern that there remains limited comprehensive national data describing critical care provision, workforce and utilisation in Zambia and the wider sub‐Saharan Africa region. It is important to note that critical care services in many low‐resource settings not only remain scarce but also may have limited quality, resulting in increased morbidity and mortality [6, 7]. Therefore, in the absence of national data, it is difficult to evaluate current services and work towards improving care. Consequently, this paper presents the findings from an initial cross‐sectional survey using an e‐questionnaire to assess the current state of critical care units in Zambia. This was a first step in identifying the key challenges for critical care services, and in providing data‐driven recommendations for improving critical care delivery.

Healthcare provision in Zambia is predominantly a public healthcare system, delivered through government facilities, together with publicly funded faith‐based providers integrated into the national health system. Services are funded through a mix of direct government funding and health insurance schemes, such as the mandatory government National Health Insurance Management Authority (NHIMA) system. However, it is important to note that many patients and families continue to have to pay out‐of‐pocket expenses for medications, investigations, etc. [8]. There is also an increasing private sector, which is predominately based within the capital Lusaka [9]. According to the latest MOH published figures (December 2021), Zambia had 7 fourth‐level hospitals, 7 third‐level hospitals, 36 second‐level hospitals, 100 first‐level hospitals, 62 mini hospitals, 1720 health centres and 1388 health posts [10].

## 2 Aims

The aim of this study was to undertake a nationwide survey of current hospital critical care workforce, service provision and patient case mix.

## 3 Methods

An adapted quantitative cross‐sectional survey from a previously validated study conducted in Malawi was used [11], aiming to determine the extent of service delivery, availability and the level of service capacity, based upon defined and measured indicators [12]. The use of standardised data‐collection instruments and predetermined indicators helped eliminate bias, increasing the reliability and repeatability of study results [12].

### 3.1 Study Site

The research project included 14 hospitals identified by the MOH as having functioning critical care units, providing representation across the 10 provinces of Zambia. Although it is accepted that Zambia is working to increase critical care provision, these sites do not include faith‐based (mission) or private hospitals as their critical care capacity was unknown at the time of data collection.

### 3.2 Sample Size and Sampling

Purposive sampling was used to assess the critical care services provided, and this information could only be gathered from hospitals with functional critical care units [9]. Hospitals were selected by the MOH to provide information on the infrastructure, workforce, equipment and service delivery within their critical care units. The MOH supported the study with a letter to the identified hospitals requesting their participation. Representation across all provinces with established critical care units was used to facilitate the possibility of national representation. A total of 14 hospitals were selected and approved by the MOH, thereby representing the present‐day organisation of critical care services in Zambia's public health system.

### 3.3 Inclusion and Exclusion Criteria

The inclusion criteria for this study were based on the hospitals reported by the MOH as having functioning critical care services. Selected hospitals included those with both adult and paediatric critical care capacities; given that there are only two dedicated paediatric intensive care units in Zambia, both units are required to admit critically ill children [4, 5]. To be eligible to participate in this evaluation, each hospital had to provide consent for the survey. Hospitals that declined to participate in the evaluation were excluded. Also, hospitals that did not report to the MOH were not included.

### 3.4 Questionnaire Design

The questionnaire used in this study was adapted from a cross‐sectional study conducted in Malawi study by Sonenthal et al. [11]. This instrument was considered appropriate due to the similarities between the Malawian and Zambian health systems, particularly in terms of critical care service organisation and workforce constraints. Adapting an existing, previously used questionnaire is a recognised approach for improving methodological rigour, as it allows researchers to build on tools that have already demonstrated relevance and applicability in comparable settings [12].

The survey was distributed via email using Microsoft Forms to hospitals and was informed by internationally recognised health facility assessment tools and critical care service indicators, including the WHO Health Systems Assessment Frameworks [13]. Aligning questionnaire content with established international frameworks enhances construct and content validity, ensuring that key dimensions of critical care workforce capacity and service provision are systematically captured [13]. The questionnaire covered key domains including hospital characteristics, number and type of critical care beds, weekly admissions and discharges, workforce and the availability of essential critical care equipment and services. The study was conducted over a 7‐day period in February 2025.

The 7‐day data collection period was selected to provide a snapshot of critical care admissions while remaining feasible in a resource‐constrained setting. This allowed for capture of weekday and weekend admission patterns; this reduces the risk that findings are influenced by daily fluctuations in patient volume or case mix. Short‐duration surveillance periods have been widely used in critical care epidemiology and point‐prevalence studies, particularly in low‐resource settings, where longer data collection periods may be impractical because of staffing and financial constraints [14, 15].

### 3.5 Data Analysis

Data were downloaded from Microsoft Forms and entered into the Statistical Package for the Social Sciences (SPSS) version 2025 for analysis. Prior to analysis, the dataset was cleaned to identify incomplete responses and checked for internal consistency and verification. Data cleaning and verification are essential procedures in quantitative research to reduce errors and improve data integrity [16].

### 3.6 Reliability and Validity

Reliability was maximised through the use of a standardised and structured questionnaire that was administered uniformly across all participating hospitals. Closed‐ended questions with predefined response categories minimised ambiguity, reduced measurement error and enhanced the internal consistency of responses [12, 16]. This strengthened the reliability of the instrument by limiting variations in administration across study sites.

Validity was enhanced by using health systems and critical care assessment indicators drawn from recognised international frameworks. The study relied primarily on routinely collected hospital records rather than on respondents' subjective recall, thereby reducing recall bias and improving the accuracy of the data [16]. Content validity was further strengthened through expert peer review and approval of the questionnaire by the MOH.

### 3.7 Ethical Considerations

Ethics approval was granted by the institutional research ethics committees in both Zambia (Ref no.: 6115‐2024; dated 16 December 2024) and the UK (Ref no.: 13690; dated: 6 November 2024). In accordance with the legal requirements, the study was then registered with the National Health Research Authority in Zambia. In addition, the MOH approved permission for the study to take place in public hospitals and each hospital approved for the study to be conducted.

## 4 Results

This study undertook a nationwide survey of current hospital critical care workforce, service provision and patient case mix. The survey had a 100% (*n* = 14) response rate, and all questionnaires were completed by Registered Critical Care Nurses (RCCNs).

### 4.1 Critical Care Capacity

Table 1 details the 14 hospital characteristics, including their level, their bed capacity, the critical care coverage and the staffing. The distribution of hospitals included two (14.3%) national referral hospitals, seven (50.0%) referral hospitals and five (35.7%) university/teaching hospitals. The number of beds in the hospitals varied from 120 to 1500, with a total of 8980 hospital beds and 131 critical care beds. Based on 131 beds and a national population of approximately 22 million, this approximates to 0.6 beds per 100 000 population. However, these figures need to be considered with caution because non‐Ministry facilities were excluded, and the reported beds may not all have been operational at the time of data collection.

**TABLE 1:** Number of hospital beds, critical care beds and critical care workforce.

No.	Level of facility	No. of hospital beds	No. of critical care beds	24‐h critical care intensivist coverage	No. of critical care nurses	Registered nurses (RNs)
1	National referral	500	5	Yes	10	5
2	National referral	500	6	Yes	6	6
3	Referral	1000	5	No	1	2
4	Referral	120	3	No	4	5
5	Referral	1500	10	No	5	6
6	Referral	300	12	No	4	6
7	Referral	360	20	No	5	7
8	Referral	400	25	No	6	8
9	Referral	400	4	No	6	9
10	University/Teaching	400	4	No	7	9
11	University/Teaching	500	6	Yes	10	7
12	University/Teaching	1000	5	No	11	8
13	University/Teaching	1500	20	No	16	20
14	University/Teaching	500	6	Yes	10	7
	Total	8980	131	—	101	105

### 4.2 Workforce

Only four hospitals (28.6%) provided 24‐h critical care cover by the physician anaesthetist with training in intensive care medicine. These comprised both national referral hospitals, and the two university/teaching hospitals. All hospitals reported having RCCNs; however, the numbers varied within each facility (Table 1).

### 4.3 Critical Care Support

Table 2 summarises the support services that were available. Thirteen out of 14 hospitals reported having functioning laboratory services. Twelve out of 14 hospitals had functioning radiological services. Computer tomography (CT) scanners were reported in eight hospitals; however, it was a cause for concern that in four hospitals they were not functioning. The remaining six hospitals reported not having a CT scanner on site. Twenty‐four‐hour pharmacy services were available in all hospitals.

**TABLE 2:** Hospital services.

Hospital	Type of facility	Laboratory service (24 h)	Radiology service (24 h)	CT scanner	Pharmacy (24 h)
1	National referral	No	No	No	Yes
2	National referral	Yes	Yes	Yes (not working)	Yes
3	Referral	Yes	Yes	No	Yes
4	Referral	Yes	No	Yes	Yes
5	Referral	Yes	Yes	No	Yes
6	Referral	Yes	Yes	No	Yes
7	Referral	Yes	Yes	No	Yes
8	Referral	Yes	Yes	Yes	Yes
9	Referral	Yes	Yes	No	Yes
10	University/Teaching	Yes	Yes	Yes	Yes
11	University/Teaching	Yes	Yes	Yes (not functional)	Yes
12	University/Teaching	Yes	Yes	Yes (not working)	Yes
13	University/Teaching	Yes	Yes	Yes (not working)	Yes
14	University/Teaching	Yes	Yes	Yes	Yes

### 4.4 Critical Care Admission

During a 7‐day period, a total of 121 adults and children were admitted to critical care units. The majority of patients were aged between 18 and 59 years (*n* = 60/50%), followed by paediatric patients (*n* = 48/40%) and adults aged > 60 years (*n* = 13/11%). Table 3 provides a breakdown of types of admission. The largest group of patients that needed critical care was medical (*n* = 35/29%), followed by surgical cases (*n* = 30/24.8%), and trauma cases (*n* = 24/19.8%). There was also a significant number of neuro‐surgical (non‐trauma cases) (*n* = 13/10.7%) and burn cases (*n* = 13/10.7%). The remaining six admissions (5%) were categorised as ‘other causes’ that did not meet any of the main categories.

**TABLE 3:** Critical care admissions (7‐day period).

Reason for admission	Adults, *n* (%)	Paediatrics, *n* (%)	Total, *n* (%)
Medical	28 (23.1)	7 (5.8)	35 (29.0)
Surgical	20 (16.5)	10 (8.3)	30 (24.8)
Trauma	10 (8.3)	14 (11.6)	24 (19.8)
Neuro‐surgical	7 (5.8)	6 (5.0)	13 (10.7)
Burns	4 (3.3)	9 (7.4)	13 (10.7)
Other	4 (3.3)	2 (1.7)	6 (5.0)
Total	73 (60.3)	48 (39.7)	121 (100)

### 4.5 Admission and Discharge Protocols

Among the surveyed facilities, five (35.7%) hospitals reported having written admission policies, whereas six (42.8%) had written discharge policies. All admissions/discharges were documented within a 24‐h timeframe. The majority of admissions and discharges occurred during morning hours (between 07:30 AM and 01:00 PM) accounting for 11 admissions and 12 discharges. During the afternoon hours (01:00 PM and 06:00 PM), nine admissions and 10 discharges were recorded. Out of hour shifts (06:00 PM–07:30 AM), 13 admissions and one discharge were recorded.

### 4.6 Critical Care Equipment

All hospitals reported having access to working ventilators (range 2–20) with most ventilators found at the two referral hospitals, followed by university hospitals. Similarly, all hospitals had functioning cardiac monitors (range 3–18), with most functioning equipment found at national referral hospitals.

Only one hospital had walled suction; the remaining units relied on portable suction machines. These varied in number, with one hospital reporting a single operational suction unit for the entire clinical department. Infusion pump availability varied, with one hospital reported access to only a single functioning syringe pump, and larger hospitals having more equipment. Syringe pumps were the least accessible equipment, with seven (50%) hospitals reporting no pumps, whereas the university hospitals reported having pumps between 2 and 20.

None of the hospitals used commercially available enteral nutrition formulas, with respondents identifying family supplied the nutrition feeds for three of the hospitals (21.4%). Five hospitals (35.7%) prepared their feeds in‐house; these included two national hospitals and three regional hospitals. The feeds were being prepared in the hospital's nutrition or unit kitchen. Respondents indicated that most hospitals (71.4%) reported using blended feeds, with the most common feed types being soya porridge, which accounted for 50.0%. Non‐soya cereal porridge accounted for 42.9% of hospitals' enteral nutrition.

## 5 Discussion

### 5.1 Critical Care Workforce

A shortage of critical care healthcare professionals is considered to be one of the main challenges in many low‐resource countries [11, 17]. This study has highlighted that although advances have been made, Zambia continues to face a critical shortage of intensivists and critical care nurses. With no in‐country formal intensive care training programmes, physician anaesthetists are required to cover the ICU. In Zambia, there are an estimated fewer than 30 consultant physician anaesthetists, which adds further complexity to critical care provision [18]. This is similar in other countries; for example, in Ghana, anaesthetic physicians made up the overwhelming majority of ICU medical staff [6].

In the absence of intensivists and other healthcare professionals, RCCNs in Zambia are expected to take on a greater responsibility for patient care, to supervise general nurses with limited training who work in ICU and to manage the unit on a day‐to‐day basis [4, 17]. These added responsibilities bring an increase in their already high workload, which increases the risk of burnout, which in turn can adversely affect patient safety. The documented shortfall of critical care staff in this study is not unusual in sub‐Saharan Africa; for example, a Malawian study by Sonenthal et al. [11] also found the number of ICU staff working in Malawian ICUs, which impacted on their ability to provide basic continuous monitoring, rapid response time and quality care.

Zambia's Strategic Health Plans [10] emphasise the importance of critical care development, workforce strengthening and infrastructure enhancement. Both plans highlight that resources must be distributed equitably, integrated into wider health service delivery and workforce training, retention strategies and monitoring of patient outcomes are key components for effective ICU service provision. These policy recommendations align directly with the survey results indicating gaps in intensivist coverage, uneven distribution of skilled staff and variability in ICU equipment availability.

### 5.2 Critical Care Provision

Despite the high demand for critical care services, many critical care units do not operate at their full number capacity, partly due to the availability of a skilled workforce, but also due to insufficient availability of equipment and support services that are needed for the delivery of service. The limited availability of functioning equipment remains one of the most significant hindrances to improvements in the provision of critical care services [5, 19, 20]. Sonenthal et al.'s [11] national assessment of emergency and critical care facilities in Malawi found that even though the units were attempting to give their population high‐quality health care, this could not be achieved due to the lack of equipment, radiological, laboratory and stock issues.

Access to critical care services is a challenge in low‐resource countries. Because critical care services are often located in major urban areas, patients frequently require long distance inter‐hospital transfer to access life‐saving care. This finding is supported by Kifle et al.'s [20] nationwide review of critical care services in Ethiopia, which reported critical care beds were only found in the capital city. A study in Ghana by Siaw‐Frimpong et al. [5] reported that one of the biggest obstacles facing critical care provision was access. With wards often being overcrowded, it was impossible to monitor and treat patients appropriately. This reduces the quality of care offered, limited recognition of the deteriorating patient and the incidence of complications likely to arise. This is compounded by the limited availability of critical care beds; for instance, Malawi has a critical shortage of ICUs with just 20 ICU beds for a population of 20 million. Similarly, Uganda has 0.1 and South Africa has 6.0 advanced critical care beds (and ventilators ready to provide mechanical ventilation) available per 100 000 of the population, respectively [6].

### 5.3 Patient Case Mix

As with many low‐resource countries in Africa, Zambia has a relatively young population, and in this cross‐sectional study, the majority of admissions were adult. This is also in line with WHO reports of a lower life expectancy in Zambia and availability of dedicated paediatric critical care services. This reflects the unique nature of critical care units and the dual role places additional demands on the workforce, particularly where staff may not have specialist paediatric training [21].

Wong et al. [22] reported bed utilisation in Malawi was underutilised, attributing this to the absence of an admission and discharge protocol. Thus, the limited availability of critical care beds, escalation protocols and admission/discharge policies increases the complexity of managing a critically ill patient and can adversely affect outcomes. A study in Malawi by Prin et al. [23] exploring the day of the week and time of day of critical care admissions found that many patients are only admitted during daytime hours on weekdays; they found no statistical link between time and day of admission with mortality. This limited period for admission could in part be due to the availability of senior staff, who are often more available during the daytime and may have the decision‐making skills and responsibility regarding admission and discharge in critical care units.

### 5.4 Strengths & Limitations

A key strength of this study was its nationwide scope and full participation of all selected hospitals, which enhances the credibility and representativeness of the findings. The use of a previously validated standardised data collection tool allowed for a snapshot assessment within a limited timeframe. Importantly, the findings offer a valuable baseline against which future evaluations and quality improvement initiatives can be measured.

The study has several limitations. The cross‐sectional nature of the survey restricts the ability to assess causal relationships between resource availability and patient outcomes, such as mortality or length of ICU stay. The data represent a snapshot in time and may not capture temporal variations in staffing, equipment functionality or service availability. The survey also did not include private or mission hospitals, although it is accepted that some of these may have critical facilities affecting the generalisability of findings beyond the public sector. Furthermore, the absence of patient‐level outcome data constrains the ability to directly link identified gaps to clinical effectiveness. In addition, this e‐survey questionnaire was self‐reported by each facility, which increases the risk of error and/or bias. It is acknowledged that this survey collected a broader range of variables than are reported in this paper; however, to maintain focus on the study objectives, only data directly relevant to the current analysis were included. Additional data may support future analyses and research on critical care services.

As with prior research in the region, nationwide or international collaboration study sample sizes were relatively small [19, 22]. In addition, several studies reported missing or incorrect records, preventing accurate data analysis [24, 25]. This results in the lack of standardised critical care services and records, meaning data accurate collection, analysis, interpretation and reporting must be treated with caution.

### 5.5 Implications for Practice and/or Future Research

The findings identify important implications for future critical care practice in Zambia. Ongoing workforce development should continue to be prioritised to increase the number of specialist critical care practitioners across the country. In addition, recognising the high proportion of paediatric admissions, dedicated paediatric critical care education programmes are urgently needed. Standardisation of admission and discharge protocols should be developed and evaluated to facilitate equity of access to critical care and enhance patient safety. There needs to be investment in essential critical care equipment which includes diagnostic services to address the variations in resource availability and maximise service provision. Future studies should be large scale, which incorporating longitudinal designs and patient outcome measures to better evaluate the impact of resource availability and staffing on survival and recovery.

## 6 Conclusion

The findings of this study have important implications for nursing practice, workforce planning and service delivery in Zambia. Given the limited availability of intensivists, the presence of at least one RCCN in all units represents a strong foundation; however, the inequitable distribution of skilled staff and reliance on general nurses without specialist training highlight an urgent need to strengthen workforce capacity and competency. Consequently, this supports the need for continued capacity strengthening of critical care education programmes for all members of the multidisciplinary team.

The high proportion of paediatric admissions also indicates a need for nurses to be equipped with paediatric critical care skills and access to appropriate equipment. In addition, there is an urgent need for standardisation of clinical protocols, including admission, discharge and nutrition practices, that is essential to promote consistency and quality of care across settings.

This cross‐sectional survey has provided a national snapshot of critical care services across government hospitals in Zambia. This survey represents an important starting point for ongoing quality improvement efforts. Future studies should incorporate longitudinal designs and patient outcome measures to better evaluate the impact of resource availability and staffing on survival and recovery. By reflecting on the findings and engaging key stakeholders, including policymakers, clinicians and educators, this study can serve as a catalyst for sustained improvements in ICU care in Zambia, ultimately benefiting both patients and the health system as a whole.

## Funding

This study was supported by the Research England through International Science Partnerships Fund (ISPF) (5452).

## Ethics Statement

Ethics approval for this study was granted by the University of Zambia Biomedical Research Ethics Committee (Ref: 6115‐2024; dated 18 December 2024) and the Birmingham City University Faculty of Health, Education & Life Sciences Ethics Committee (Ref: 13680/sub3/R(C)/2024/Oct/HELS FAEC; dated 5 November 2024). In addition, the study was registered with the National Health Research Authority in Zambia (Ref: NHRAR‐R‐2326/20/12/2024; dated 23 December 2024), and the study was formally registered with the Ministry of Health in Zambia for implementation within public healthcare facilities.

## Conflicts of Interest

The authors declare no conflicts of interest.

## Data Availability

The data that support the findings of this study are available on request from the corresponding author. The data are not publicly available due to privacy or ethical restrictions.
